# Two Cases of Rare Intratumoral Hemorrhage Following COVID-19 Vaccination

**DOI:** 10.7759/cureus.32400

**Published:** 2022-12-11

**Authors:** Shin Sugasawa, Toshikazu Kimura, Yuan Bae, Toshio Kumasaka, Shunsuke Ichi

**Affiliations:** 1 Stroke and Cerebrovascular Surgery, Sagamihara Kyodo Hospital, Sagamihara, JPN; 2 Neurological Surgery, Japanese Red Cross Medical Center, Tokyo, JPN; 3 Pathology, Japanese Red Cross Medical Center, Tokyo, JPN

**Keywords:** neurosurgery cyberknife, meningioma, vestibular schwannoma, covid-19 vaccine, intratumoral hemorrhage

## Abstract

The increase in the rate of mRNA vaccination against coronavirus disease 2019 (COVID-19) worldwide has been accompanied by reports of an increase in the side effects of the vaccine. In the field of neurosurgery, several cases of venous thrombosis have been reported as possible complications after COVID-19 vaccination. However, no such side effects have been reported in patients with brain tumors, and COVID-19 vaccination is considered safe for this patient population. In this report, we present the cases of two patients with brain tumors who experienced intratumoral hemorrhage as a possible side effect of the COVID-19 vaccine. In the first case, a 54-year-old man who had received CyberKnife treatment for a vestibular schwannoma eight years prior presented with tongue discomfort, right-side facial numbness, and dizziness since the day after his COVID-19 vaccination. MRI revealed intratumoral hemorrhage of the vestibular schwannoma. The second patient was a 60-year-old woman who presented with a sudden-onset headache and vomiting that had started three days after her COVID-19 vaccination. CT revealed a meningioma with intratumoral hemorrhage. Both patients had undergone surgery prior to this presentation, and their symptoms had improved. They had no risk factors for intratumoral hemorrhage, suggesting that it may be a side effect of the mRNA vaccine against COVID-19. Although the causal relationship is unclear, acute inflammation with predominantly lymphocytic infiltration and thrombogenicity after COVID-19 vaccination may damage the fragile microcirculation of the tumor.

## Introduction

The efficacy and safety of vaccines against coronavirus disease 2019 (COVID-19) have been vigorously debated. Although myocarditis and other side effects have occasionally been reported, reports on the vaccine's neurosurgical side effects have been scarce, except for venous thrombosis in some individuals [1]. In this report, we present two cases of intratumoral hemorrhage following COVID-19 mRNA vaccination.

## Case presentation

Case 1

**Figure 1:**
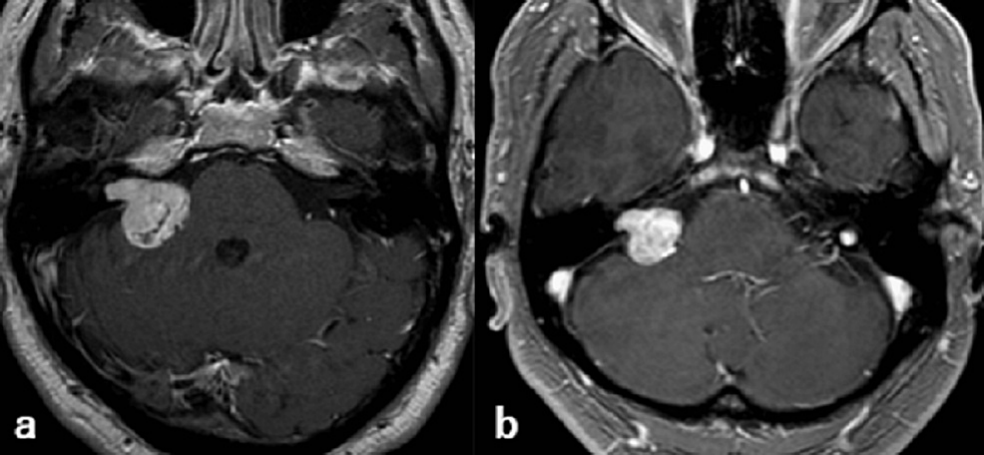
MRI findings - (a) before treatment (b) six years after CyberKnife treatment MRI: magnetic resonance imaging

A 54-year-old man with no significant medical history other than Caisson’s disease presented with tongue discomfort, facial numbness, and dizziness since the day after getting his COVID-19 vaccination (Moderna-mRNA-1273, third dose). He had undergone CyberKnife^Ⓡ^ (Accuray, Sunnyvale, CA) stereotactic radiosurgery (SRS) for Koos grade 3 right acoustic neuroma eight years prior. MRI at his last follow-up six years after the CyberKnife treatment had shown a slight shrinkage of the tumor (Figure 1).

**Figure 2:**
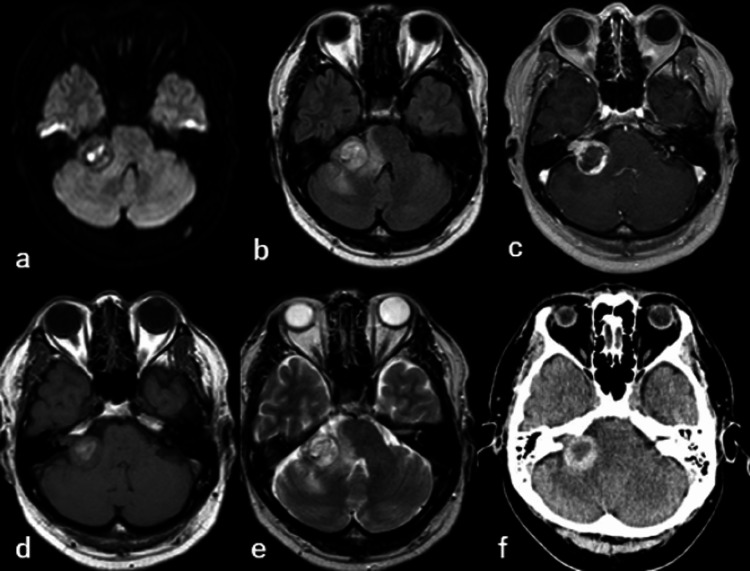
Imaging findings - (a) DWI (b) FLAIR (c) gadolinium-enhanced (d) T1 (e) T2 (f) CT DWI: diffusion-weighted image; FLAIR: fluid-attenuated inversion recovery; CT: computed tomography

The patient's consciousness was clear, with right-sided deafness and grade II right-side facial palsy according to House-Brackmann (HB) grading, with occasional spasms of the orbicularis oculus muscle. He also complained of sensory disturbance in the right half of his tongue and altered taste. Cerebellar ataxia, such as gait disturbance or paralysis of the limbs, was not observed. Brain MRI showed an increase in tumor size. The intensity of the tumor had changed, showing high intensity on diffusion-weighted images and T1-weighted images, and heterogeneity on T2-weighted images, which suggested intratumoral hemorrhage. High-intensity signals were prominent in the brainstem and cerebellar peduncle on fluid-attenuated inversion recovery (FLAIR) images. T2-weighted images showed that the tumor was compressing the trigeminal nerve. The tumor showed a heterogeneous density on CT (Figure 2).

**Figure 3:**
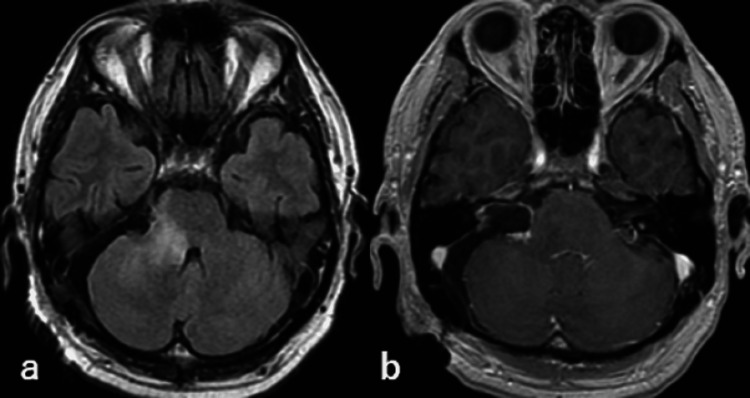
MRI findings on the third postoperative day - (a) FLAIR (b) gadolinium-enhanced MRI: magnetic resonance imaging; FLAIR: fluid-attenuated inversion recovery

Because the patient’s symptoms were consistent with the MRI findings, the tumor was surgically removed. An old hematoma was observed in the cistern around the tumor. The tumor itself consisted of typical necrotic tissue after radiation and partially viable schwannoma tissue with a hematoma at its center. Gross total resection was performed, leaving a small portion of the tumor around the facial nerve (Figure 3).

Postoperatively, the patient's facial numbness and dizziness while walking improved. The facial nerve palsy improved to HB grade I. The patient was discharged with a modified Rankin Scale (mRS) score of 1. At a follow-up visit two months after the surgery, the facial palsy had subsided.

**Figure 4:**
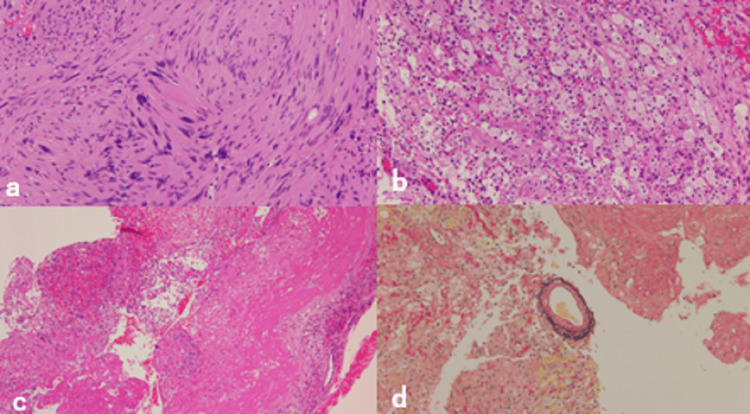
Pathological findings - (a) Antoni A region (b) Antoni B region (c) necrotic tissue and hemorrhagic components (d) EVG staining EVG: Elastica van Gieson

The pathological findings were typical of a vestibular schwannoma, and there was no mitotic image suggestive of malignancy. Elastica van Gieson staining revealed no obvious thrombi in the specimen. The surrounding necrotic tissue with hemorrhage showed infiltration by foam cells and lymphocytes (Figure 4).

Case 2

A 60-year-old woman with no significant medical history started experiencing a sudden-onset severe headache and vomiting three days after getting the first dose of COVID-19 vaccination (Pfizer-BioNTech, BNT162b2). Because her symptoms persisted even after five days, she visited our clinic.

**Figure 5:**
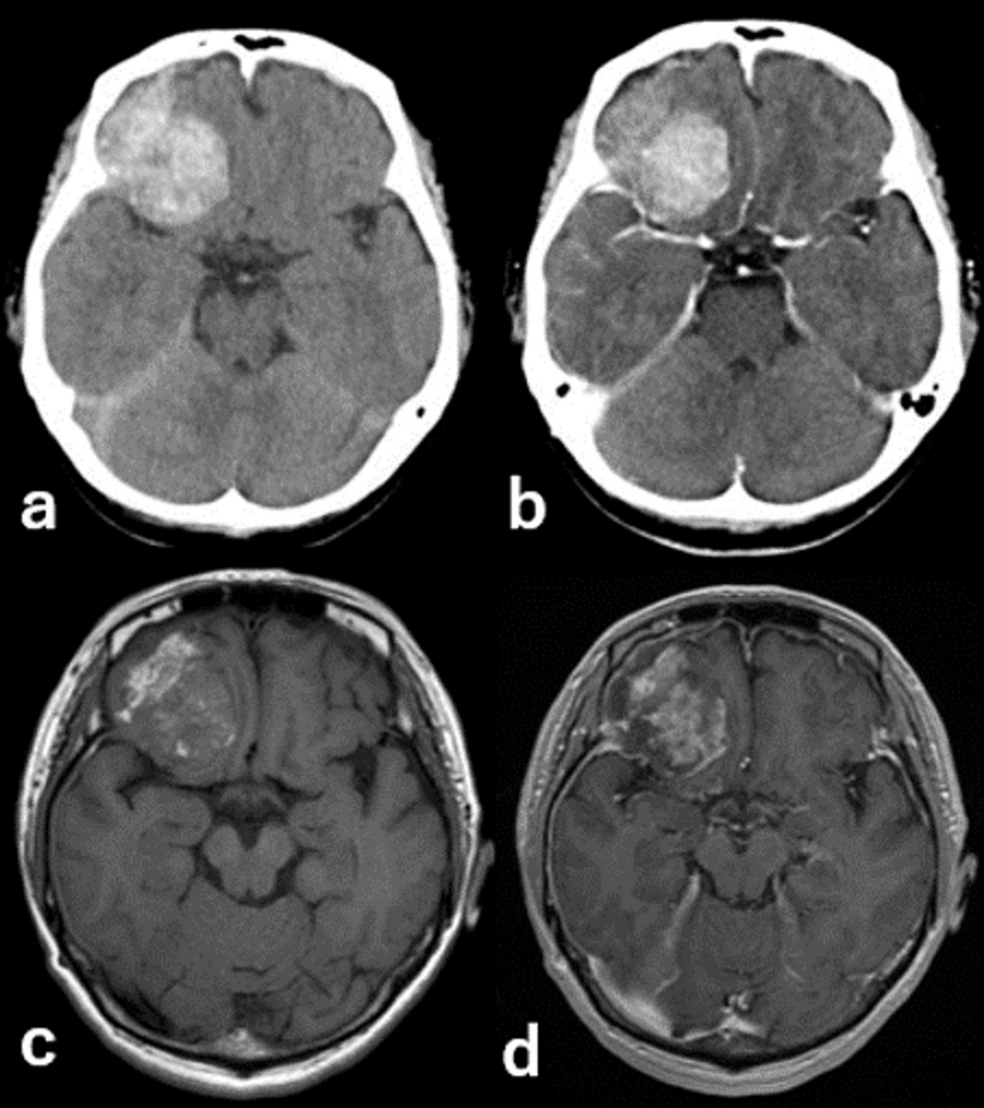
Imaging findings - (a) plane CT (b) enhanced CT (c) T1 (d) enhanced MRI CT: computed tomography; MRI: magnetic resonance imaging

She had a clear consciousness and no obvious neurological symptoms other than the headache. Brain CT showed a high-density mass in the right anterior cranial fossa and ipsilateral subdural hematoma, and contrast-enhanced CT showed a uniform contrast effect. There was no prominent high-intensity signal around the mass on FLAIR, and the lesion was enhanced homogeneously with gadolinium-diethylenetriaminepentaacetic acid (Gd-DTPA) (Figure 5).

**Figure 6:**
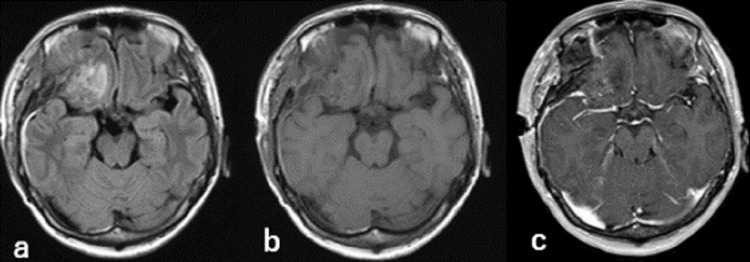
Imaging findings six days after the surgery - (a) FLAIR (b) T1 (c) enhanced MRI FLAIR: fluid-attenuated inversion recovery; MRI: magnetic resonance imaging

The patient underwent craniotomy for tumor resection under general anesthesia. Both the tumor and its dural attachment site were resected, as the pathology indicated a meningioma. Postoperatively, the patient developed partial right oculomotor nerve palsy, probably due to the excision of the dura mater through the superior orbital fissure. The patient was discharged on postoperative day 19 with an mRS score of 2 (Figure 6).

**Figure 7:**
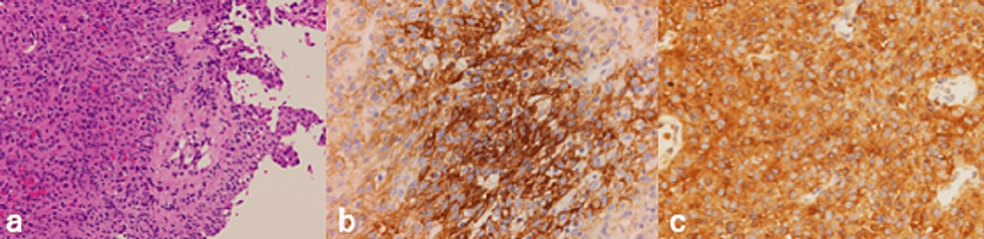
(a) HE staining (b) EMA staining (c) vimentin staining HE: hematoxylin and eosin; EMA: epithelial membrane antigen

The pathological diagnosis revealed World Health Organization (WHO) grade I meningothelial meningioma. The tumor cells had oval nuclei and eosinophilic spores, with indistinct cell borders. Partial hemorrhage, inflammatory cell infiltration, and granulation were observed; however, no necrosis or infiltration of the brain parenchyma was observed. Immunostaining showed epithelial membrane antigen (EMA)-positive, vimentin-positive, and CD34-negative staining. The dura mater of the tumor attachment showed negative margins (Figure 7).

## Discussion

We presented two cases of intratumoral hemorrhage immediately following mRNA vaccination for COVID-19. Various side effects of COVID-19 mRNA vaccines have been reported. Among these, there have been several cases of venous thrombosis [2]. The period of symptom onset after vaccination is 4-19 days in cases of venous thrombosis [1].

In case 1, intratumoral hemorrhage of a vestibular schwannoma, which was stable for eight years after SRS, occurred on the day after vaccination. Intratumoral hemorrhage in vestibular schwannomas is rare, with a reported incidence of 0.4% in general [3] and 0.26% after SRS [4]. It usually occurs 80 or 130 months after SRS [4,5]. Although the mechanism of intratumoral hemorrhage and the effects of the radiation are not completely understood, anticoagulation has been cited as a risk factor for intratumoral hemorrhage in vestibular schwannomas [3]. The histological presentation of this case was similar to that of previous cases of vestibular schwannoma with intratumoral hemorrhage, showing pathologically foamy macrophages and surrounding necrotic tissue [6,7]. In addition, similar to myocarditis after vaccination, acute inflammation with predominantly lymphocytic infiltration was observed pathologically [8,9]. Kawashima et al. have reported that microhemorrhage or thrombosis due to vascular endothelial damage is likely to cause intratumoral hemorrhage [4].

In case 2, meningioma with intratumoral hemorrhage resulted in symptoms of headache and vomiting three days after the vaccination, and the tumor was EMA-positive and vimentin-positive pathologically, consistent with WHO grade I meningioma [10]. Meningiomas are one of the most common benign tumors [11]. Intratumoral hemorrhage in meningiomas is very rare, with a reported prevalence of 0.5-2.3% [12] or 2.2% [13]. Additionally, 67.9% of hemorrhagic meningiomas have been reported to be located in the convexity region, 50% presenting as intracerebral hemorrhage, and 27.3% as subdural hematoma [14]. The cause of intratumoral hemorrhage is unclear; however, it is thought to be caused by disruption of tumor vessels, disruption of bridging veins due to growing tumors [13], and venous infarction due to venous obstruction with cerebral edema [15]. Moreover, previous studies have reported that meningiomas with intratumoral hemorrhage are often WHO grade I [13,14] and that the risk factors for intratumoral hemorrhage include age >70 years, hypertension, anticoagulant therapy, trauma, serotonin regulation therapy, and high-volume estrogen replacement therapy [16].

In both cases, COVID-19 vaccination appeared to have affected the tumor to cause bleeding. In case 1, the patient presented with various neurological symptoms following the third dose, while the female patient in case 2 had a headache after the first dose. One study has reported myocarditis complicated by the booster dose [17], but its mechanism and other details remain unclear. In addition, there have been no other reports of increased side effects due to booster doses. Takeyama et al. have recently reported a case of intracerebral hemorrhage due to vasculitis with an autoimmune reaction mediated by the vaccine [18]. However, their findings on disease pathology contrast with those in our two cases. Both patients had no risk factors for the reported intratumoral hemorrhage, indicating it to be a side effect of the mRNA vaccines against COVID-19. Although the causal relationship is unclear, it is possible that acute inflammation with predominantly lymphocytic infiltration along with damage to the microcirculation due to thrombus formation may have led to intratumoral hemorrhage.

The number of reports on the side effects of COVID-19 vaccines has increased with the increase in the number of vaccinations worldwide. AstraZeneca and Johnson & Johnson vaccines causing vaccine-induced thrombocytopenia caused by antiplatelet factor IV antibodies has been reported [1]. The COVID-19 vaccine is generally considered effective and safe in patients with brain tumors. According to the findings from a single cancer institute in Italy, 102 patients with brain tumors who received the vaccination, including 100 who received a second dose and 73 who received the booster dose, experienced no side effects [19]. Based on an online survey conducted in 42 countries, Voisin et al. reported that none of the 965 patients with brain tumors experienced serious side effects from any vaccine [20]. Considering the number of people who received COVID-19 mRNA vaccines in our country and the prevalence of meningioma and vestibular schwannoma, the side effect presented in our two cases should be considered a rare complication. Therefore, it can be speculated that some predisposing factors may have contributed to hemorrhage in our cases. However, we should not rule out the possibility that increased mRNA vaccination thrombogenicity and acute inflammation with predominantly lymphocytic infiltration may have damaged the fragile microcirculation of the tumor.

## Conclusions

We discussed two cases of intratumoral hemorrhage in benign tumors following mRNA vaccination against COVID-19. Intratumoral hemorrhage can occur due to acute inflammation with predominantly lymphocytic infiltration or fragile microcirculation with thrombus formation. The causal relationship between mRNA vaccines and the occurrence of intratumoral hemorrhage requires further investigation. Further studies are needed to determine the frequency of intratumoral hemorrhage following mRNA vaccination.
